# External Quality Assessment for Tuberculosis Diagnosis and Drug Resistance in the European Union: A Five Year Multicentre Implementation Study

**DOI:** 10.1371/journal.pone.0152926

**Published:** 2016-04-07

**Authors:** Vladyslav Nikolayevskyy, Doris Hillemann, Elvira Richter, Nada Ahmed, Marieke J. van der Werf, Csaba Kodmon, Francis Drobniewski, Sabine Ruesch-Gerdes, ERLTB-Net Network

**Affiliations:** 1 PHE National Mycobacterium Reference Laboratory, London, United Kingdom; 2 National Reference Laboratory for Mycobacteria, Forschungszentrum Borstel, Borstel, Germany; 3 European Centre for Disease Prevention and Control, Stockholm, Sweden; 4 Imperial College London, London, United Kingdom; Universidad Nacional de la Plata, ARGENTINA

## Abstract

**Background:**

External quality assurance (EQA) systems are essential to ensure accurate diagnosis of TB and drug-resistant TB. The implementation of EQA through organising regular EQA rounds and identification of training needs is one of the key activities of the European TB reference laboratory network (ERLTB-Net). The aim of this study was to analyse the results of the EQA rounds in a systematic manner and to identify potential benefits as well as common problems encountered by the participants.

**Methods:**

The ERLTB-Net developed seven EQA modules to test laboratories’ proficiency for TB detection and drug susceptibility testing using both conventional and rapid molecular tools. All National TB Reference laboratories in the European Union and European Economic Area (EU/EEA) Member States were invited to participate in the EQA scheme.

**Results:**

A total of 32 National TB Reference laboratories participated in six EQA rounds conducted in 2010–2014. The participation rate ranged from 52.9% - 94.1% over different modules and rounds. Overall, laboratories demonstrated very good proficiency proving their ability to diagnose TB and drug-resistant TB with high accuracy in a timely manner. A small number of laboratories encountered problems with identification of specific Non-tuberculous Mycobacteria (NTMs) (N = 5) and drug susceptibility testing to Pyrazinamide, Amikacin, Capreomycin, and Ethambutol (N = 4).

**Conclusions:**

The European TB Reference laboratories showed a steady and high level of performance in the six EQA rounds. A network such as ERLTB-Net can be instrumental in developing and implementing EQA and in establishing collaboration between laboratories to improve the diagnosis of TB in the EU/EEA.

## Introduction

Tuberculosis (TB) remains an important public health problem globally and within the European Union (EU) despite significant progress made in the past decade. In 2013, 64 844 TB cases were reported in 28 EU and two European Economic Area (EEA) countries, 6% less than in 2012 and reflecting a decrease in 19 countries [1]. TB notification rates remained high (>40.0/100,000) in three countries (Latvia, Lithuania, and Romania) with the highest incidence estimated in Romania (87.0/100,000) (1). Although there has been a steady decrease in TB notification rates both in EU/EEA countries and the World Health Organisation (WHO) European Region as a whole over the last decade (1), the target of 50% reduction of TB prevalence by 2015 set up by The Stop TB Partnership, which is linked to the Millennium Development Goals, is unlikely to be achieved. Despite the low percentage of multidrug-resistant (MDR) TB in the EU/EEA region (4.1%), the proportion of MDR TB remained high in Latvia, Lithuania, and Estonia (11.6, 18.9, and 22.7%, respectively) [1].

Timely and accurate diagnosis of active TB is a prerequisite for any successful TB control programme and an essential part of the action framework to eliminate TB in low-incidence countries [2]. The laboratory plays a key role in TB diagnosis both at individual and programmatic level through detection of active TB cases and drug susceptibility testing (DST), contributing to contact tracing and surveillance through highly discriminatory genotyping as well as latent TB infection diagnosis [3–7]. In addition, detection of drug resistance is crucial for administering optimal treatment regimens and prevention of transmission.

To ensure compliance with existing international standards of laboratory diagnostics [8], all laboratories in the EU should be accredited by the relevant national bodies. Adequate laboratory infrastructure and facilities as well as appropriately trained and qualified personnel and both Internal and External Quality Assurance (IQA and EQA) systems are essential for obtaining and retaining accreditation [9]. Participation of TB diagnostic laboratories in various EQA schemes organised by international bodies and recognised providers has proven effective in maintaining and improving laboratory proficiency across the EU/EEA and elsewhere [10–12].

The introduction of new drug susceptibility testing methods, including rapid culture and molecular tools, and genotyping methodologies necessitated the development of novel as well as modification of existing EQA schemes (eg for rapid molecular tools). A series of recent reports revealed specific problems related to a lack of standardisation in second-line drug (SLD) susceptibility testing and multilocus MIRU-VNTR genotyping [13–15]. The development and implementation of advanced EQA schemes can identify these problems early and address them through networking activities and collaboration at national and supranational level.

A situation analysis of national TB reference functions across the EU [3] formed the basis for launching the European Reference Laboratory Network for TB (ERLTB-Net, formerly ERLN-TB) in January 2010. The network was established to meet the objectives and strategies described in the `Framework Action Plan to Fight Tuberculosis’ [16]. The network is co-ordinated by the European Centre for Disease Prevention and Control (ECDC) and a consortium of partners led by Public Health England (PHE). It aims to consolidate and strengthen TB laboratory capacity, improve quality and achieve sustainability in TB laboratory diagnosis through provision of training, harmonisation of laboratory methods, development and implementation of reliable EQA systems and standards in TB laboratory diagnosis within the EU/EEA and beyond as well as supporting the functionality of national TB laboratory networks. The development of a sustainable EQA system constitutes one of the major network activities. The developed system has been implemented through regular EQA rounds and in-depth analysis of the results, addressing identified challenges through training, task-force visits and other mechanisms. Initial insights into the results of proficiency testing for DST were published in 2013 [15].

The aim of this study was to systematically analyse the implementation of the EQA scheme across the network from 2010 to 2014, assess performance of the laboratories, identify common problems and discuss the potential benefits of the scheme for the participants.

## Materials and Methods

### Participating laboratories and study design

A total of six EQA rounds were conducted from 2010 to 2014 (Table 1). All 34 National TB Reference laboratories (NRL) nominated by relevant national bodies were invited to participate in the EQA. Panels of specimens (except crude DNA extracts for Module 7) were prepared at the NRL Germany, shipped to INSTAND e.V. (an independent non-profit interdisciplinary scientific medical society, nominated as a Collaborating Centre for Quality Assurance and Standardisation in Laboratory Medicine of the World Health Organisation since 1994) and distributed across the ERLTB-Net network. Crude DNA extracts were prepared at the PHE National Mycobacterium Reference Laboratory (NMRL) and distributed via INSTAND e.V. The test results of the EQA samples were sent back to INSTAND e.V. by the participating laboratories. Scoring and analysis was done jointly by INSTAND e.V. and NRL Germany and individual results were reported back to the participants. Participation in EQA rounds was voluntary and free of charge for all participating laboratories. All laboratories ensured safe working conditions and participated only in the modules covering procedures performed routinely in their laboratories.

**Table 1:** External Quality Control modules and specimen panel composition for six rounds, 2010–2014.

EQA modules		Round 1 Spring 2010	Round 2 Autumn 2010	Round 3 2011	Round 4 2012	Round 5 2013	Round 6 2014
1	Microscopy	6 slides	6 slides	6 slides	6 slides	6 slides	6 slides
2	Primary Isolation	5 spiked sputum specimens	5 spiked sputum specimens	5 spiked sputum specimens	5 spiked sputum specimens	5 spiked sputum specimens	5 spiked sputum specimens
3	Identification	5 *M*. *spp* strains	5 *M*. *spp* strains	5 *M*. *spp* strains	5 *M*. *spp* strains	5 *M*. *spp* strains	5 *M*. *spp* strains
4	Drug susceptibility testing	5 *M*. *tuberculosis* strains	5 *M*. *tuberculosis* strains	10 *M*. *tuberculosis* strains	10 *M*. *tuberculosis* strains	5 *M*. *tuberculosis* strains	5 *M*. *tuberculosis* strains
5	Rapid NAAT identification of Mycobacteria	Not performed	Not performed	5 spiked sputum specimens	5 spiked sputum specimens	5 spiked sputum specimens	4 spiked sputum specimens
6	Molecular DST (cultures)	Not performed	5 *M*. *tuberculosis* strains	10 *M*. *tuberculosis* strains	10 *M*. *tuberculosis* strains	10 *M*. *tuberculosis* strains	5 *M*. *tuberculosis* strains
7	Molecular DST (crude extracts)	Not performed	Not performed	6 crude DNA extracts	6 crude DNA extracts	Not performed	Not performed

EQA, External Quality Control. NAAT, Nucleic Acid Amplification Tests. DST, Drug Susceptibility Tests. *M*. *spp*, various Mycobacterium genus species

### EQA modules

The ERLTB-Net developed seven modules covering various types of laboratory techniques: (1) microscopy; (2) primary isolation; (3) identification; (4) drug susceptibility testing; (5) nucleic acid amplification testing (NAAT) for rapid detection of Mycobacteria; (6) molecular drug susceptibility testing using cultures; and (7) molecular drug susceptibility testing using crude DNA extracts (Table 1).

Artificial sputa (spiked with mycobacteria) were used for preparation of slides (for microscopy) and specimens for primary isolation and rapid NAAT-based detection; all specimens in the latter panels contained equal concentrations of Mycobacteria. For identification and DST, strains with validated characteristics (*Mycobacterium* species and drug susceptibility profiles) were used. Specimen panels included strains with varying DST profiles (S1 Table); no MDR strains were included due to safety concerns. Crude DNA extracts for Module 7 were prepared using heat-lysed *M*. *tuberculosis* suspensions treated with chloroform [17]; among these non-hazardous samples, DNA isolated from MDR strains was included (S2 Table). Module 3 panels comprised of *M*. *tuberculosis* complex (MTBC) strains (*M*. *tuberculosis*, *M*. *bovis BCG*. *M*. *bovis* subsp *caprae*, *M*. *bovis* subsp *bovis*) as well as non-tuberculous mycobacteria (NTM) (*M*. *gordonae*, *M*. *kansasii*, *M*. *fortuitum*, *M*. *abscessus*, *M*. *szulgai*, *M*. *intracellulare*, *M*. *celatum*, *M*. *chelonae*, *M*. *avium*, *M*. *marinum*, *M*. *simiae*, *and M*. *malmoense*) in various combinations.

### Methods used in participating laboratories

The laboratories used their routine methods to test the specimens provided within the EQA panels. The laboratories were asked to report on the methods used for smear microscopy, rapid detection of Mycobacteria (Modules 1 and 5) and (optionally) methods used for species identification (Module 3).

### Data analysis and laboratory certification

Reports received from participating laboratories were analysed against reference results as determined by the EQA provider (NRL Germany and INSTAND e.V), and the percentage of correct results in each module was calculated by dividing the number of correct results by the total number of tests in the module. This provided the score for the given laboratory in the given module. If not all tests within a given module were performed (for example, for second line DST), the overall score was adjusted accordingly. Laboratories scoring ≥80% were issued performance certificates for individual modules. Their individual performances were referred to as “good” (scores 80.0…99.9%) and “excellent’(100.0%) and overall performance of laboratories achieving 80% score was collectively termed “good”. Scores under 80% were considered to indicate poor performance for the specific module(s) and no EQA certificate was issued [15].

Error rates for specific methods were calculated as a proportion of incorrect results relative to a total number of individual tests performed by all laboratories across all rounds; for example in Modules 5 and 6 data was analysed by individual drugs. Proportions were compared using the chi-square test using GraphPad Prizm (La Jolla, California, USA). A p-value < 0.05 was considered as significant.

## Results

**Fig 1:**
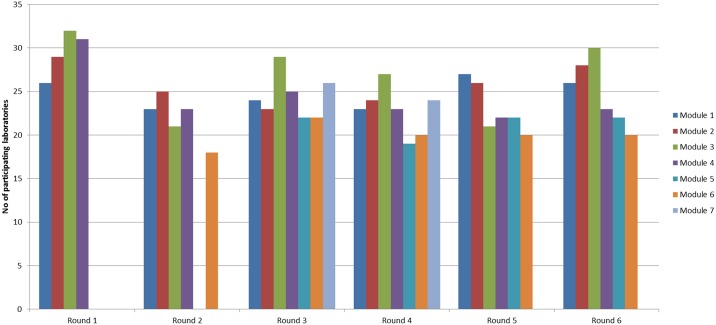
Laboratories participating in EQA rounds and modules.

The number of participating laboratories varied across EQA rounds and modules with the largest number of laboratories (N = 32) participating in the *Mycobacterium* species identification module in round 1 comprising 94.1% of laboratories within the network (Fig 1). These 32 laboratories represented 27 EU/EEA Member States (five countries have more than one laboratory with NRL status).

**Fig 2:**
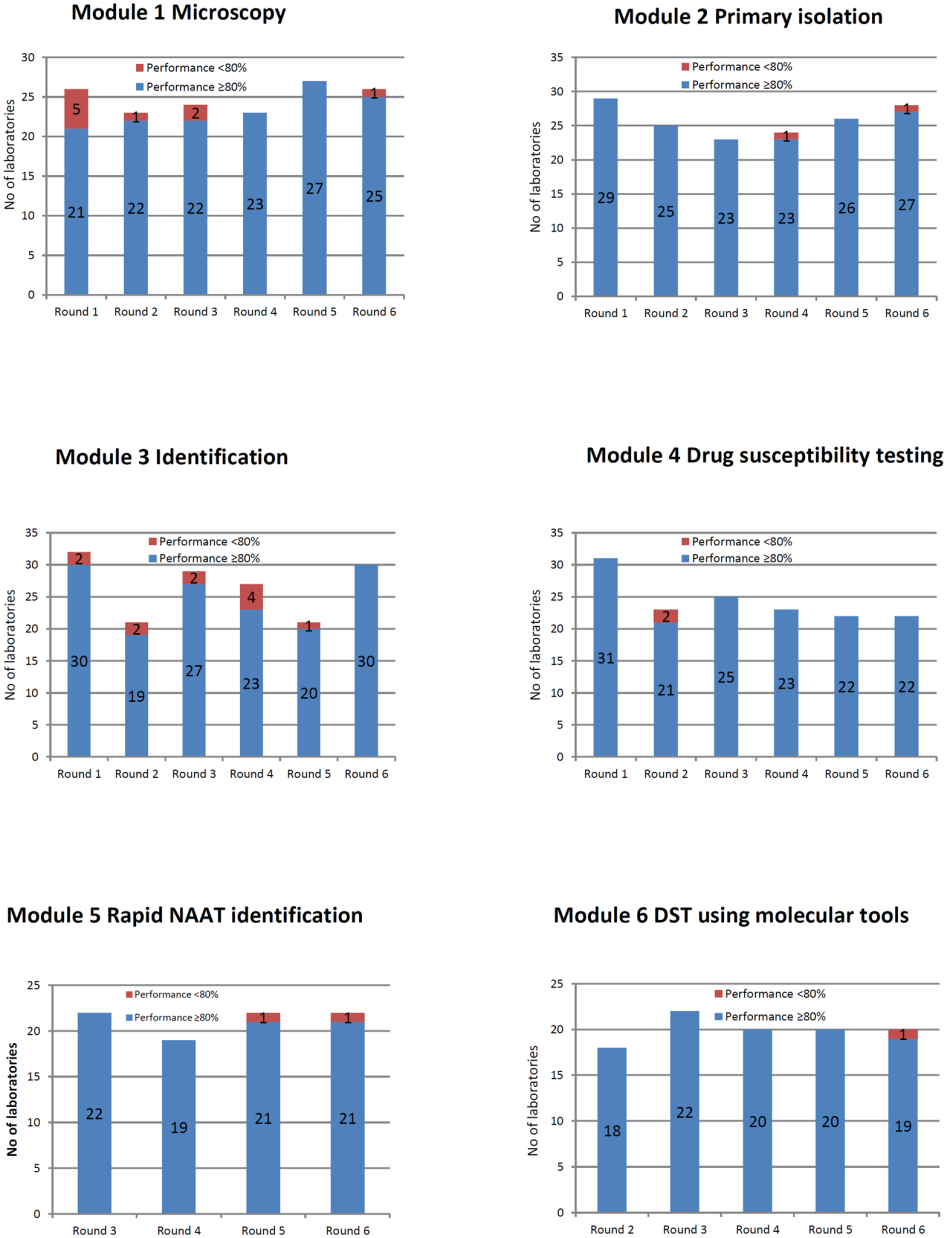
Results of proficiency testing in rounds 1 to 6 by module.

Performance characteristics of participating laboratories in the EQA rounds 1–6 are summarised in Fig 2.

### Module 1 – Microscopy

The proportion of laboratories using Ziehl-Neelsen (ZN) staining for smear microscopy (Module 1) varied from 46.0% to 58.0% with the remaining laboratories using primarily auramine fluorescent staining; a small number of laboratories (varying from 1 to 4 in different rounds) used the Kinyoun method.

**Fig 3:**
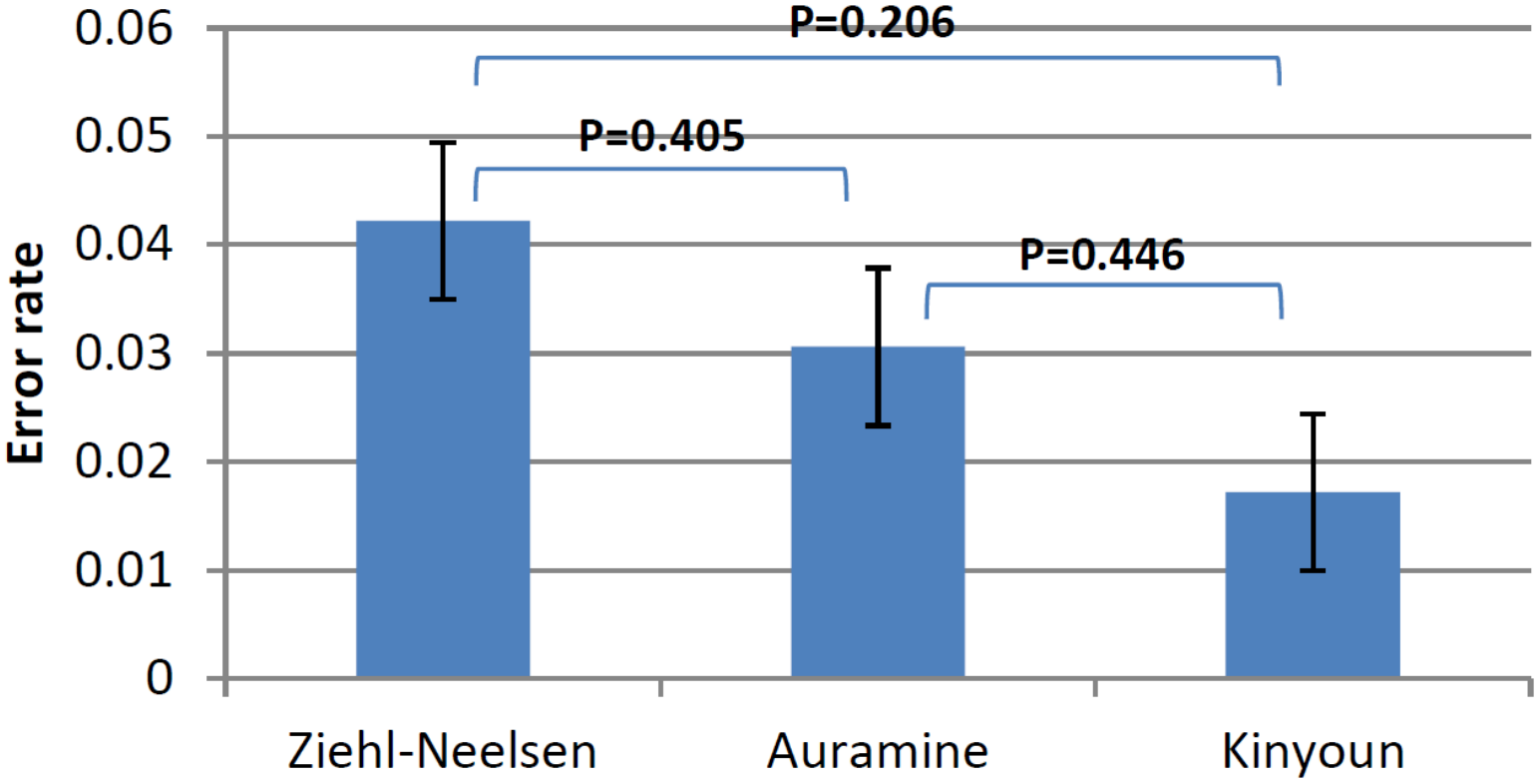
Smear microscopy error rates for microscopy diagnosis by Ziehl-Neelsen, auramine or Kinyoun methods.

There were minor variations in the numbers of laboratories participating in the proficiency testing for microscopy in different rounds with the largest number (N = 27) taking part in round 5 and the smallest (N = 23) in rounds 2 and 4. In rounds 2–6, laboratories demonstrated good and excellent performance with only a small number (one in round 2, two in round 3, zero in round 4 and 5, and one in round 6) of laboratories failing to achieve the 80% threshold. The proportion of laboratories that were awarded certificates was 80.8% in round 1 and 96.2% in round 6. There were no significant differences in error rates and overall performance characteristics between laboratories using different smear staining methods (ZN, auramine, or Kinyoun) (Fig 3).

### Module 2 – Primary isolation

Within module 2, laboratories were requested to detect the presence of mycobacteria in artificial spiked sputum specimens using bacteriological culture, and (if routinely performed) differentiate *M*. *tuberculosis* complex from NTM and identify *Mycobacteria* species using a range of phenotypic and/or molecular tools. The number of participating laboratories varied from 23 (round 3) to 29 (round 1). Overall scores were good and excellent with only one laboratory in rounds 4 and 6 failing to achieve the 80% threshold. The proportion of laboratories reporting results of species identification varied from 64.3% to 95.7% in different years and largely depended on the composition of the EQA panels (MTBC or NTM) as not all laboratories identify the same spectrum of NTM species routinely on primary specimens. Common problems encountered by the laboratories included reporting the presence of mycobacteria in negative specimens (probably indicating cross-contamination, N = 5), and inability to detect NTM in positive specimens (N = 2).

### Module 3 – Species identification

In module 3, participants performed species identification of mycobacterial cultures using phenotypic or molecular tools or a combination of both based on standard protocols adopted in those laboratories. The number of participating laboratories ranged from 21 (Rounds 2 and 5) to 32 (Round 1).

Within this module, performance varied considerably with a total of three laboratories across all rounds achieving low scores of 20–40% (in rounds 1, 2, and 4) and one laboratory misidentifying all specimens in round 5. The majority of participating laboratories, however, demonstrated either good or excellent performance. The proportion of laboratories that were awarded certificates varied from 85.2% (round 4) to 100% (round 6). Common problems experienced by the laboratories included misidentification of NTM and misidentification of species within the *M*. *tuberculosis* complex (a total of 24 incorrect NTM identifications and 16 incorrect identifications within the *M*. *tuberculosis* complex in all rounds) with *M*. *bovis caprae* and *M*. *bovis* BCG being the most problematic species.

### Module 4 – Phenotypic drug susceptibility testing

In module 4, laboratories were requested to test five (modules 1–2 and 5–6) or ten (modules 3 and 4) *M*. *tuberculosis* cultures for sensitivity to five first-line drugs (FLD) (all modules) and additionally to fluoroquinolones (FQ), capreomycin (CAP), amikacin (AMK), and kanamycin (KAN) (Modules 3–5) or FQ, CAP, and AMK (Modules 2 and 6) using any validated phenotypic method routinely used in the laboratory. All but one laboratory used automated liquid culture-based systems (Bactec MGIT960, Becton Dickinson, New Jersey, USA) for DST for second line drugs; for FLD including PZA methods varied and included Bactec MGIT960 as well as the proportion method on solid media and resistance ratio method on semi-solid and liquid media for PZA.

The number of laboratories taking part in module 4 varied from 22 (Rounds 5 and 6) to 31 (Round 1) and overall performance was good with only two laboratories failing to achieve 80% in one round (round 2). Only susceptibility to rifampicin (RIF) and isoniazid (INH) was tested by all participating laboratories; other drugs were tested by a smaller number of laboratories in accordance with their routine practices. Importantly, the proportion of laboratories routinely testing for susceptibility to pyrazinamide (PZA), FQ and AMK increased over the five years from 65.0%, 56.6%, and 52.2% to 100.0%, 77.3%, and 77.3%, respectively, confirming the ability of many laboratories to detect extensively drug-resistant (XDR) strains.

**Fig 4:**
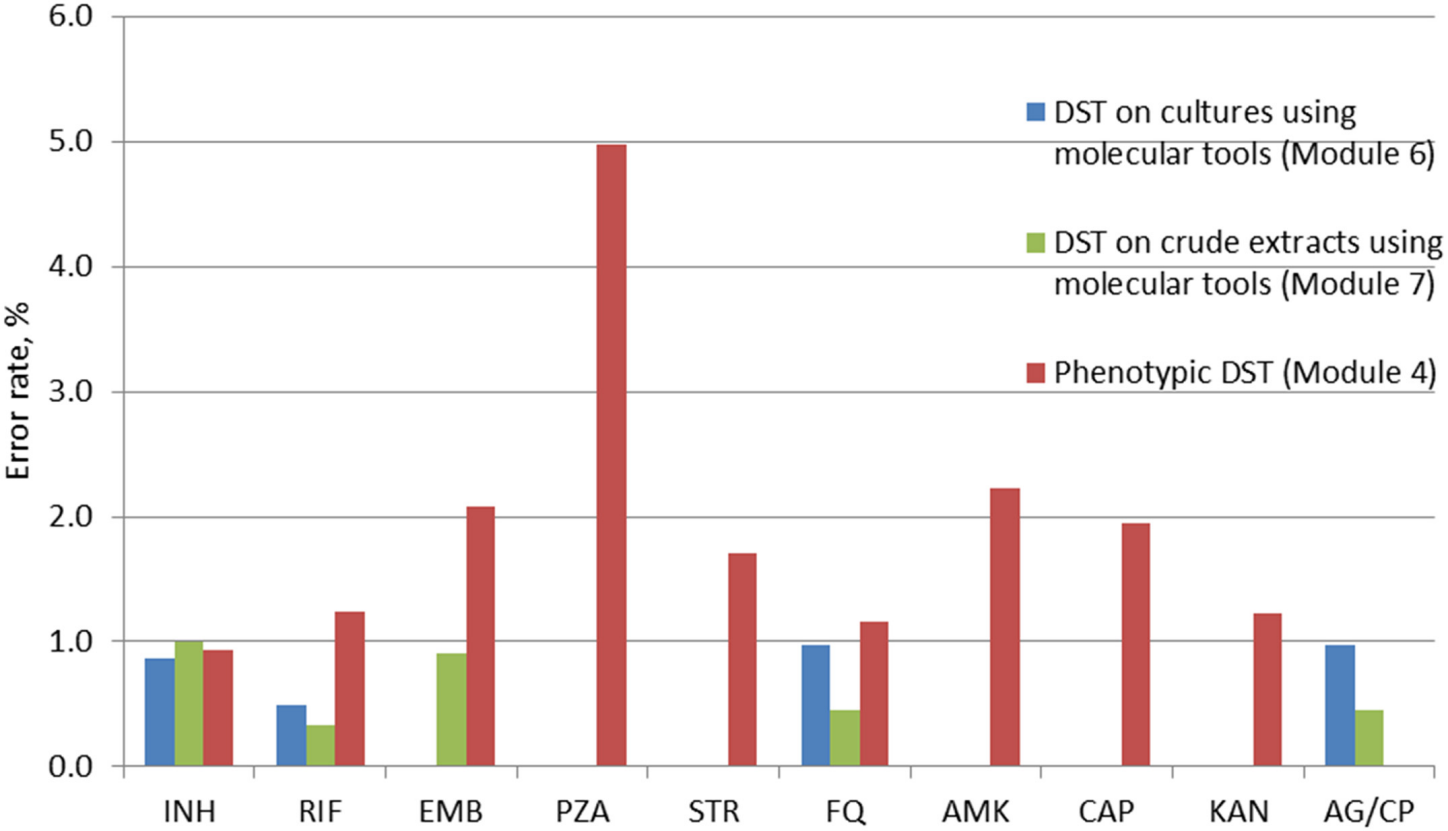
Error rates for tuberculosis drug susceptibility testing by phenotypic tools and molecular tools grouped by individual drugs. INH, isoniazid; RIF, Rifampicin; EMB, Ethambutol; PZA, pyrazinamide; STR, streptomycin; FQ, fluoroquinolones; AMK, amikacin; CAP, capreomycin; KAN, Kanamycin; AG/CP, aminoglycosides/cyclic peptides. DST, Drug Susceptibility Testing. For molecular tests, a combined resistance to injectable drugs (“AG/CP”) was reported instead of individual resistance to AMK, CAP, and KAN.

Error rates varied substantially between drugs (Fig 4). PZA was consistently the most problematic drug (41 incorrect results comprising 5.0% of all drug susceptibility tests) followed by AMK, ethambutol (EMB) and streptomycin (STR) (13, 20 and 11 errors comprising 2.2%, 2.1%, and 1.9%, respectively). The proportion of incorrect results for other second-line drugs (FQ and KAN) was lower and similar to that for INH and RIF.

### Module 5 – Rapid identification of mycobacteria using molecular tools

This module was designed to test proficiency of laboratories in rapidly detecting MTB complex bacteria in sputum specimens using nucleic acid amplification tests (NAAT). The number of participating laboratories ranged from 19 (Round 4) to 22 (remaining rounds) and performance was good with all but two laboratories (in Rounds 5 and 6) achieving scores >80%. The proportion of laboratories using GeneXpert (Cepheid, Sunnyvale, USA) methodology for TB detection in primary specimens increased from 35.0% in 2011 to 65.2% in 2014. Other methods used for TB detection in primary specimens included BD ProbeTec DTB (Becton Dickinson, New Jersey, USA) used by up to 13.6% of laboratories in different years, and Cobas TaqMan (Roche, Pleasanton, USA) GT Mycobacteria Direct (Hain Lifescience GmbH, Nehren, Germany), and Artus PCR kit (QIAGEN GmbH, Hilden, Germany) (all used by less than 10% of the laboratories).

There were no differences in error rates and overall performance characteristics between GeneXpert and other NAAT methods for rapid MTB complex bacteria identification.

### Module 6 – Molecular DST on cultures

In Module 6, proficiency of laboratories in rapidly detecting resistance to selected first (INH, RIF, and EMB) and second-line drugs (FQ like Oxofloxacin or Moxifloxacin, and the injectables AMK, KAN, and CAP) on cultures was tested. The number of participants remained relatively stable over five years and ranged from 18 (Round 1) to 22 (Round 2). Nearly all laboratories used line probe assays (LPAs) for molecular DST. A small number of laboratories (<10%) used in-house validated assays including targeted gene sequencing and pyrosequencing,

Performance was good with only one laboratory in round 6 failing to achieve the 80% threshold score. The proportion of laboratories adopting technologies for rapid DST for second-line drugs increased from 66.7% (round 2) to 85.0% (round 6). Overall concordance with reference results was high, exceeding 99% for all drugs (Fig 4). Unlike the phenotypic tests, there was no significant variation and error rates were generally very low (<1.0%) for both first and second-line drugs (Fig 4) demonstrating excellent proficiency of participating laboratories in rapid DST and overall feasibility of molecular tests.

### Module 7 – Molecular DST on crude extracts

The primary aim of this module was to test the proficiency of the laboratories in rapid detection of highly drug-resistant *M*. *tuberculosis* strains, and therefore inactivated non-hazardous crude DNA extracts isolated from fully sensitive, poly- and multidrug-resistant strains were used. Module 7 panels were only included in rounds 3 and 4 and results were obtained from 26 and 24 laboratories, respectively.

Overall performance was good with a total of 8 errors reported (comprising 0.6% of all tests performed within the module). All laboratories achieved scores >80%. Errors were not confined to any specific drug and were lower for RIF (0.3%) and higher for INH (1.0%) (Fig 4). Concordance with the reference values was >99% for all drugs and groups of drugs. Since performance of all participating laboratories in Module 7 in two consecutive years was excellent, it was decided to discontinue Module 7 to minimise associated shipping and laboratory costs.

## Discussion

The primary aim of this study was to systematically analyse the performance of national TB reference laboratories for detection, identification and drug susceptibility testing for TB diagnosis across the EU/EEA through a comprehensive series of EQA rounds, identify common problems and discuss the potential benefits of the EQA scheme developed within the EU-wide TB reference laboratory network.

Overall, laboratories demonstrated good proficiency with scores consistently exceeding the 80% threshold in the vast majority of the laboratories. Steady high performance in both phenotypic and rapid molecular methods over five years with only 27 individual module result failures (3.4% of the total) shows an ability of national TB Reference laboratories to diagnose TB and drug resistance TB with high accuracy in a timely manner. The principal results of our study were in agreement with another report [15] demonstrating the sustainability in TB diagnostic laboratories’ performance achieved over the last decade through activities of several collaborative projects including Nordic-Baltic TB Network, FP7 TB PANNET and other initiatives [18,19].

Challenges with microscopy and species identification in rounds 1–3 have been addressed through corrective actions including additional training and laboratory visits. No major problems were detected in primary isolation (module 2). An in-depth analysis of laboratory performance for phenotypic DST has allowed us to identify methodological problems with specific anti-TB drugs including PZA, EMB and, interestingly, KAN and AMK. This is largely in agreement with earlier reports demonstrating a lack of standardised methodologies and drug concentrations for PZA (type of media) and KAN (use of mono- or disulphate salts) [15,19,20] and highlights the importance of developing novel and/or improving existing methods for phenotypic DST for certain drugs It also shows the advantages of molecular tools as their laboratory performance did not depend on the drug tested in our study and agreement rates with reference results were generally better compared to phenotypic tests. Overall performance of phenotypic DST for key drugs (RIF, INH, FQ) was good and demonstrated the capacity of most participating laboratories to reliably detect MDR and XDR TB. Noteworthy, the quality of TB detection in our study did not depend on the method used. This suggests that reproducibility and performance characteristics of established and novel methods in microscopy and identification of mycobacteria do not vary greatly, providing adequate quality control measures are in place.

Proficiency of laboratories in identification and DST using rapid molecular tools proved to be good and excellent. High scores were achieved by the laboratories in molecular DST on cultures and crude extracts and there was a significant increase in the number of laboratories adopting molecular techniques (particularly the GeneXpert system), which is critical for the timely detection of MDR strains and prevention of transmission [4,5].

Our study identified several common challenges in EQA schemes with relatively low participation rates being one of them. Significant variability of participation rates (52.9–94.1%) across modules and/or EQA rounds can be in part explained by the fact that not all laboratories perform all the tests routinely (e.g. microscopy, primary isolation or second line DST); data collected outside the current study suggests that up to 25% of NRLs are not routinely engaged in primary laboratory work (unpublished data). A small number of laboratories encountered challenges in sending the results within the agreed timeframe or were unable to take part due to financial constraints (although the EQA itself was free for all participating laboratories). This highlights the importance of adequate management and careful financial planning including allocation of funds for participation in EQA schemes. Other problems encountered in individual laboratories preventing them achieving the 80% threshold included clerical errors (mislabelling etc), incorrect interpretation of molecular and phenotypic tests results, as well as cross-contamination issues. These problems may be indicative of more general problems in the laboratory practice and should be taken into account in the laboratory quality management.

## Conclusions

Laboratory services play a crucial role in improving the delivery of health care and reducing the prevalence of TB and drug-resistant TB in particular. Quality assessment through proficiency testing is a fundamental tool used to ensure accuracy of test results by comparing quality between laboratories, evaluating performance and detecting errors so that corrective actions, including additional training and support visits, can be taken in a timely manner to restore the high quality of services.

Overall, results of our study and earlier reports on proficiency testing and EQA schemes for *M*.*tuberculosis* genotyping [13,14] demonstrated that networking activities such as through the ERLTB-Net can develop and implement a sustainable EQA system and maintain high quality TB laboratory diagnosis. Regular free EQA rounds have proven essential in establishing the current performance and identifying strengths and challenges in laboratory diagnosis of TB in the EU/EEA. The EQA scheme developed within ERLTB-Net project could be expanded to national TB laboratory networks since the EQA constitutes an essential component of the support activities provided by NRLs to regional laboratories.

Participating laboratories benefited from the developed EQA program in a number of ways including free of charge EQA and performance certificates essential for their accreditation by relevant national bodies, availability of training for their staff members as well as targeted actions including direct consultations with the EQA providers and support visits from scientists within the network who were able to provide additional expertise and help to NRLs where needed. Steadily high performance during the implementation period suggests that aforementioned network activities addressing the challenges identified through the analysis of EQA results were effective. Sustainability of EQA and training activities at EU/EEA level as well as their further development through inclusion of novel validated methodologies (eg Next Generation Sequencing) are crucial for ensuring timely and high-quality laboratory diagnosis of TB in the EU/EEA.

## Supporting Information

S1 Table Composition of the Module 4 EQA panels. (DOCX)

S2 Table Composition of the Module 7 EQA panels. (DOCX)
