# Supplementary material for: External Quality Assessment for Tuberculosis Diagnosis and Drug Resistance in the European Union: A Five Year Multicentre Implementation Study
Source: PLoS One. 2016 Apr 7;11(4):e0152926. doi: 10.1371/journal.pone.0152926 (PMC4824391; doi:10.1371/journal.pone.0152926)
Supplement: S1 Table — (DOCX) [file pone.0152926.s001.docx]

**S1 Table**

Composition of the Module 4 EQA panels

| EQA Round/  Specimen No | Expected results/sensitivities | | | | | | | | |
| --- | --- | --- | --- | --- | --- | --- | --- | --- | --- |
|  | INH | RIF | EMB | PZA | STR | FQ | AMK | CAP | KAN |
| Round 1  Specimen 1  Specimen 2  Specimen 3  Specimen 4  Specimen 5 | S  S  R  S  S | R  S  S  R  S | S  S  S  S  S | S  S  R  S  S | S  S  S  R  S | -  -  -  -  - | -  -  -  -  - | -  -  -  -  - | -  -  -  -  - |
| Round 2  Specimen 1  Specimen 2  Specimen 3  Specimen 4  Specimen 5 | R  S  S  S  S | S  S  R  S  S | S  S  S  S  S | S  S  S  S  S | S  S  R  S  - | S  S  S  S  S | S  S  S  S  S | S  S  S  S  S | -  -  -  -  - |
| Round 3  Specimen 1  Specimen 2  Specimen 3  Specimen 4  Specimen 5  Specimen 6  Specimen 7  Specimen 8  Specimen 9  Specimen 10 | S  R  S  S  R  S  S  R  S  S | R  S  S  S  S  S  R  S  R  S | R  S  S  S  S  S  S  S  S  S | S  S  S  S  R  S  S  S  S  S | S  S  S  S  S  S  R  R  S  S | S  S  S  S  S  S  S  S  S  R | S  S  S  S  S  S  S  S  S  S | S  S  S  S  S  S  S  S  S  S | S  S  S  S  S  S  R  S  S  S |
| Round 4  Specimen 1  Specimen 2  Specimen 3  Specimen 4  Specimen 5  Specimen 6  Specimen 7  Specimen 8  Specimen 9  Specimen 10 | R  S  R  S  S  S  S  S  S  S | S  R  S  S  S  S  S  S  S  S | S  R  S  S  S  S  S  S  S  S | S  S  R  S  S  S  S  S  S  S | S  S  S  S  S  S  S  S  S  S | S  S  S  S  S  R  S  S  S | S  S  S  S  S  S  S  S  R  R | S  S  S  S  S  S  S  S  R  R | S  S  S  S  S  S  S  S  R  R |
| Round 5  Specimen 1  Specimen 2  Specimen 3  Specimen 4  Specimen 5 | S  S  S  S  R | S  S  S  R  S | S  S  S  R  S | S  S  S  S  S | S  S  S  S  S | S  S  S  S  R | S  S  R  S  S | S  S  R  S  S | S  S  R  S  S |
| Round 6  Specimen 1  Specimen 2  Specimen 3  Specimen 4  Specimen 5 | S  R  S  R  S | S  S  R  S  S | S  S  S  S  S | S  S  S  S  S | S  S  S  S  S | S  R  S  S  S | R  S  S  S  S | R  S  S  S  S | -  -  -  -  - |
