# Supplementary material for: External Quality Assessment for Tuberculosis Diagnosis and Drug Resistance in the European Union: A Five Year Multicentre Implementation Study
Source: PLoS One. 2016 Apr 7;11(4):e0152926. doi: 10.1371/journal.pone.0152926 (PMC4824391; doi:10.1371/journal.pone.0152926)
Supplement: S2 Table — (DOCX) [file pone.0152926.s002.docx]

S2 Tablerbc

Composition of the Module 7 EQA panels

| EQA Round/  Specimen No | Expected results/sensitivities | | | | |
| --- | --- | --- | --- | --- | --- |
|  | INH | RIF | EMB | FQ | AG/CP |
| Round 3  Specimen 1  Specimen 2  Specimen 3  Specimen 4  Specimen 5  Specimen 6 | S  R  R  R  R  R | S  R  R  R  R  R | S  S  R  S  S  S | S  S  S  R  S  S | S  S  S  S  S  S |
| Round 4  Specimen 1  Specimen 2  Specimen 3  Specimen 4  Specimen 5  Specimen 6 | R  S  S  R  R  R | R  S  S  R  R  R | R  S  S  S  S  S | S  S  S  S  R  S | S  S  S  S  S  R |
